# An exploratory study on differences in maternal care between two ecotypes of Nigerian indigenous chicken hens

**DOI:** 10.3389/fvets.2022.980609

**Published:** 2022-09-28

**Authors:** Victor J. Oyeniran, Oluwaseun S. Iyasere, Samuel O. Durosaro, Fasasi B. Fasasi, Peace O. Odetayo, Sulaiman A. Ogunfuyi, Paul O. Odetunde, Taiwo C. Akintayo, James O. Daramola

**Affiliations:** ^1^Department of Animal Physiology, Federal University of Agriculture, Abeokuta, Nigeria; ^2^Albrecht Daniel Thaer-Institut für Agrar-und Gartenbauwissenschaften Tierhaltungssysteme und Ethologie, Berlin, Germany; ^3^Department of Animal Breeding and Genetics, Federal University of Agriculture, Abeokuta, Nigeria; ^4^Department of Animal Sciences, Purdue University, West Lafayette, IN, United States

**Keywords:** behavior, broodiness, ecotype, fear, maternal care, Nigerian indigenous chickens

## Abstract

The Yoruba (YRE) and Fulani (FLE) are the two notable indigenous chicken ecotypes in Nigeria. They exhibit broodiness and post-hatch care of their chicks. Studies on welfare, productivity, and maternal behaviors of these two ecotypes are scarce, hence the need for this study. Separate flocks of these ecotypes were housed intensively and hens that showed broodiness (ten YRE and five FLE) were monitored. Brooding behaviors were monitored for 3 days in the 1st and 2nd weeks of brooding and daily in the 3rd week of brooding for 6 h/day (07:00–09:00 h, 11:00–13:00 h, and 15:00–17:00 h). During brooding, surface body temperatures (eye, brood patch and under the wings), egg temperature and body weight of the hens were measured. Chicks hatched (44 chicks from the YRE and 24 chicks from the FLE) by these hens were subjected to tonic immobility tests on the 7th, 14th, and 21st days post-hatch and to a simulated predator test on the 8th, 15th, and 22nd days post-hatch to determine their level of fear. In each ecotype, brooding behaviors did not change over the three weeks, but the YRE hens spent longer time sitting on their eggs at the 2nd (*U* = 5.000, *z* = −2.454, *P* = 0.014) and 3rd (*U* = 9.000, *z* = −1.961, *P* = 0.050) week of brooding. The surface body temperatures of both ecotypes, egg temperature, and relative weekly weight loss were similar over the brooding period, but relative weekly weight loss was greater (*P* < 0.05) at the 3rd than 1st and 2nd week of brooding. The surface body temperatures were positively correlated (*P* < 0.01) with egg temperature. In both ecotypes, attempts to induce and duration of tonic immobility were similar over the test periods but on the 7th day post-hatch, the duration of tonic immobility was longer (*U* = 323.000, *z* = −2.632, *P* = 0.008) and on the 14th day post-hatch, the number of attempts to induce tonic immobility was less (*U* = 332.000, *z* = −2.630, *P* = 0.009) in the YRE chicks. In conclusion, YRE hens sat more on the eggs and their chicks were more fearful.

## Introduction

In developing and underdeveloped countries, indigenous chickens are more abundant, with Nigeria having the most among the Sub-Saharan countries (1). Nigerian indigenous chickens (NICs) are found in several geopolitical zones around the country and are classified according to genetic lines of feathering (normal feather, naked neck, and frizzle feather), color variants (black, white, brown, and mottled), and ecotypes [Yoruba (YRE) and Fulani (FLE)] (2, 3). Both ecotypes are good scavengers and have excellent immunity against endemic diseases (4). They are known for their hardiness, adaptability and survivability (5).

According to the Food and Agriculture Organization (6), ecotype refers to a population within a breed that is genetically adapted to a specific habitat. The natural habitat where these two chicken ecotypes are prevalent differs. The FLE is found in the dry savannahs (Guinea and Sahel savannah) while the YRE is found in the forest zones (7). However, due to the settlement of Fulani herdsmen and their families in the forest zone, where they can get forage for their cattle, they brought their chickens (FLE) with them. Presently, there is an increasing population of FLE in the forest zone. Some people in the Southwest part of Nigeria prefer to buy and raise the FLE over the YRE ecotypes, probably because of their bigger size for more meat, higher egg production and better feed conversion.

**Figure 1:**
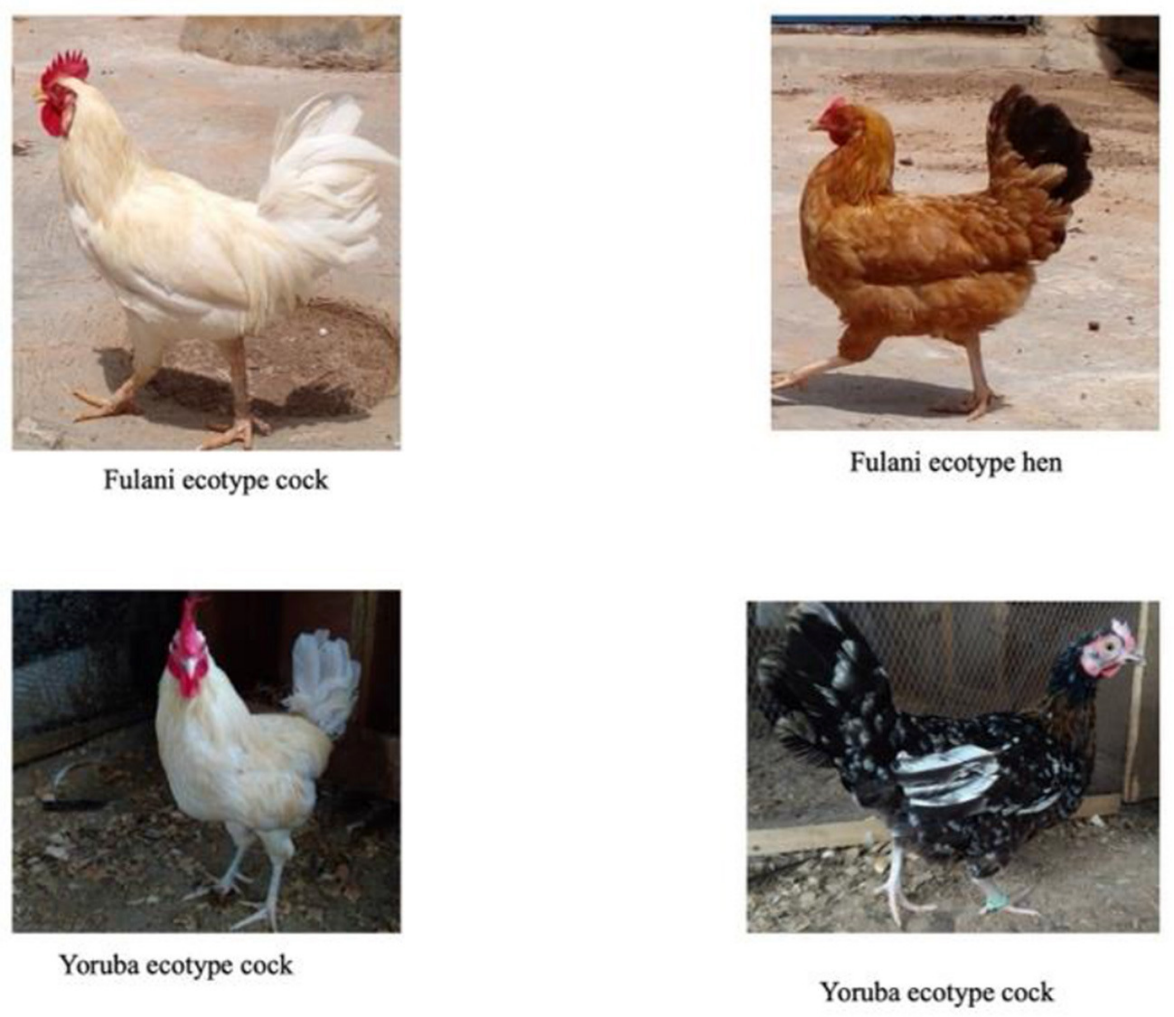
Fulani and Yoruba ecotype chickens.

There are reports on the differences between the YRE and FLE in terms of body weight, body structure, and egg production capacities, but little is known about their maternal behavior. The FLE weighs between 1.2 and 2.0 kg at maturity (8–10) while the YRE weighs between 0.68 and 1.50 kg at maturity (11). Based on body structure, the FLE and YRE are referred to as the “heavy ecotype” and “light ecotype”, respectively (12) (Figure 1). The chest circumference, wingspan, beak length, tarsometatarsus length, and body length of the FLE are greater than the YRE (13). In terms of egg production, the YRE lays earlier (20–23 weeks) than the FLE (22–31 weeks), but the FLE lays bigger, and more eggs compared to the YRE (14). These chicken ecotypes can serve as a rapid means of bridging protein deficiency and providing an additional source of income to the livelihoods of low-income families in urban, peri-urban, and rural settlements (15). Thus, these chickens play major roles in rural economies and contribute significantly to the Gross National Product of Nigeria (16).

Since these two chicken ecotypes are reared under the scavenging system, selecting an ecotype with good productivity and mothering abilities will benefit the poultry industry. In the first, second, and third weeks of brooding, YRE hens spent 88–93% and 0.06–0.11% of their time sitting on the eggs and engaging in ingestive behavior, respectively (17). The YRE hens showed behavior indicative of distress (increased pacing) when separated from their chicks visually rather than physically (18).

The indigenous chickens still exhibit their full natural behavior repertoire, which is very important to animal welfare (19). However, genetic selection for increased egg production in commercial laying hens has eliminated broodiness (20), which means that these hens can neither incubate eggs nor hatch chicks by themselves. Although chicks are precocial animals, they still require maternal care, especially in the first few weeks of life (21), to survive in the natural environment. In commercial poultry production, chicks can survive without their mothers, but this comes with several welfare issues. Rearing without a mother hen has major effects on the chicks' behavioral development (22). Brooded birds are less fearful at a young age (23), show greater exploratory behavior in a new environment (24, 25), and display less feather pecking and cannibalism, resulting in lower mortality rates compared to non-brooded birds (26).

Commercial laying hens have serious welfare issues such as feather pecking and cannibalism. The occurrence of this behavior has been linked to the lack of maternal care in early life. Hewlett and Nordquist (27) found no effect of maternal care in a commercial hybrid line of layer hen (a cross between White Leghorn and the Brown Nick), probably because the selection process has impaired the response of these chicks to maternal care. The style of maternal care adopted in their study was a cross-fostering type (using a Silkie Bantam hen to foster the commercial laying chicks). Both chicken breeds have different behavioral repertoires and welfare issues. The hybrid layer chicks already have their own innate behavior which they have inherited from their parents, which is different from that of the foster mother.

Although commercial strains may not show maternal care when reared by mothers, even after 45 weeks of removal from the mothers, the hens showed changes in brain structures (an increase in arginine vasotocin neurons in the medial pre-optic area of the hypothalamus), suggesting that they were receptive to maternal care. This indicates that commercial strains can only benefit from maternal care but cannot be maternal caregivers (28). Maternal caregiving can be found only in chickens that have not been subjected to genetic selection for increased egg productivity.

With the increasing impact of climate change on animal welfare, selecting an ecotype that is already adaptable to the tropical environment with high production capacity to meet the required protein needs (egg and meat) of the Nigerian population is needed. So, we aimed to identify the ecotype with better mothering abilities to raise chicks of good welfare with the potential to escape from predators and survive in the natural environment or free-range housing system. To achieve this aim, we assessed the brooding behavior of the two ecotypes and the longer-term effects of maternal care on fear of the offspring of these ecotypes using the conventional tonic immobility (TI) and a simulated predator test. We also examined whether the fear level of the chicks increased as they age. We hypothesized that there would be differences in the brooding behavior of the two ecotypes due to the differences in their genetic make-up which has conferred on them different body sizes, structure, and productivity. This in turn will reflect in some behavioral differences in the fear level of their chicks.

## Materials and methods

### Experimental site

The experiment was carried out at the Poultry Unit of the Directorate of University Farms (DUFARMS), Federal University of Agriculture, Abeokuta (FUNAAB). The University is located on latitude 7°10'N, longitude 3°2'E, and altitude 76 m above sea level. The area lies in the Southwestern part of Nigeria and has a prevailing tropical climate with a mean annual rainfall of 1,037 mm and an annual mean temperature and relative humidity of 28°C and 82%, respectively.

### Experimental birds and management

All procedures in this study were based on guidelines of the Animal Care and Use Committee of the Federal University of Agriculture, Abeokuta, Ogun State, Nigeria. Thirty hens and five cocks per ecotype (YRE and FLE) were selected for this study. The FLE cocks and hens used in this study had an average weight of 2 and 1.2 kg, respectively, while the YRE cocks and hens had an average weight of 1.4 and 0.8 kg, respectively.

The two ecotypes were housed each in five separate deep litter floor pens (3 × 5 m) littered with 5 cm of wood shavings. In each replicate pen, a cock and six hens were housed and provided with a perch (111 cm high), nest boxes (30 × 30 cm), and sand-bath (76 × 76 × 5 cm) that allowed the birds to perform their natural behaviors. The FLE chickens were obtained from a Fulani settlement at Kishi, Oyo State, Nigeria, and allowed to acclimatize for a month before the commencement of the experiment. The YRE chickens were obtained from an already existing flock at the research station. Once broodiness was confirmed (continuous sitting on eggs), the hens were separated into brooding (BRD) pens (similar in size to their home pens) and 10 eggs (laid by hens of the same ecotype) were placed underneath them in a nest box. The nest boxes were bedded with 2 cm of wood shavings to prevent the eggs from breaking. All the birds were provided with ready-made layer mash having the following composition: 16.5% CP, 2,725–2,980 kcal/kg metabolizable energy, 5% fat/oil, 6% crude fiber, 3.60% calcium, 0.45% available phosphorus, 0.80% lysine, 0.34% methionine and 0.30% salt. Birds were fed this compounded feed at 120 g/bird/day and water was provided *ad libitum*.

### Experimental procedure

#### Surface body and egg temperatures

The surface body temperatures (SBTs) of the broody hens were measured three times a week and their body weights were measured weekly. The SBTs of the hens were measured from three body parts (eye, under the wing, and brood patch) using a non-contact infra-red thermometer (Model: IT-122, accuracy ± 0.2°C, made in China). Also, the temperature of the eggs (EGT) was measured using an infra-red thermometer and the average egg temperature was calculated.

#### Brooding behavior

The behaviors of the brooding (BRD) hens (10 YRE and 5 FLE) were recorded for three weeks. Each BRD hen was monitored three times weekly during the first two weeks of BRD, and then daily during the last week of BRD for a total of six hours/day (morning = 07:00–09:00 h, afternoon = 11:00–13:00 h, and evening = 15:00–17:00 h) using CCTV cameras (Winposse, Model: WP-F6036TP-H, lens 3.6 mm, made in China) with 2.0 Megapixels, positioned to cover the entire pen. The behaviors of interest include sitting on the egg, turning of eggs, feeding, drinking, vigilance with eyes open, and eyes close while sitting on the eggs, as described in Table 1. After hatching, the nest box, unhatched eggs, and broken shells were removed from the pen, and the hen and her chicks were left in the same pen until the fourth-week post-hatch (PTH) when the chicks were weaned. The chicks were provided with chick mash (CP = 21%, metabolizable energy of 3,000 kcal/kg) in chick tray feeders (diameter 20 cm) and water in bell drinkers (diameter of 21.50 cm, 2-l capacity). Each chick was wing-tagged after hatching for easy identification.

**Table 1:** Behavioral categories and description.

**Behavior category**	**Description**
Sitting on eggs	Hen sitting continuously on the egg
Turning of eggs	Hen turns the egg with her beak intermittently or moves her body gently against the egg
Feeding	Hen leaves nesting position and directs its beak into the feed trough and starts pecking at the feed
Drinking	Hen leaves nesting position and directs its beak into the bowl drinker to drink water
Eyes open while sitting on eggs	Hen maintains nesting position with the eye opened
Eyes close while sitting on eggs	Hen maintains nesting position but intermittently closes the eye

#### Tonic immobility test (TI)

The level of fear in the chicks was measured using the TI test. To assess the level of fear, forty-four YRE and twenty-four FLE chicks were tested at each time (7, 14, and 21 st-day PTH) between 9:00 and 11:00 h. The chicks were chosen at random from their mothers and tested individually in a separate test room within the same poultry house by restraining them for 15 s with one hand on the sternum and the other on the head and placing them on a table. Then both hands were released. The variables observed were the number of attempts to induce TI and the latency of the bird to righting itself i.e., duration of TI was recorded with a stopwatch (maximum duration was 5 mins). If the immobility duration was <10 s before the chick righted itself, the induction was considered unsuccessful and the chick was subjected to another TI test and the number of attempts was recorded. Longer durations of TI are interpreted as indicating a higher level of fear (29). Immediately after testing each chick, it was returned to its mother and the next chick was picked.

#### Predator test

**Figure 2:**
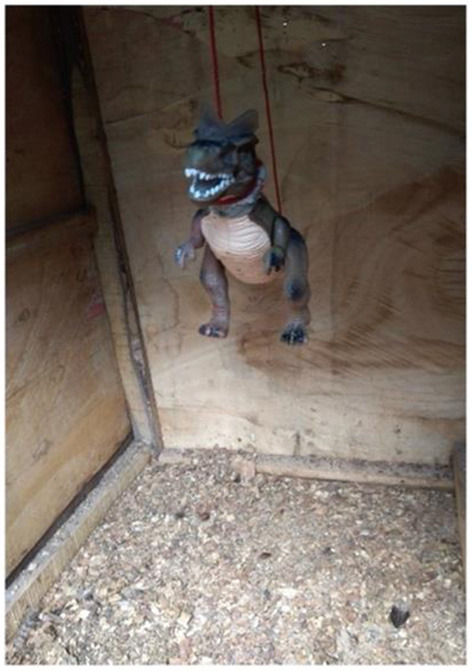
Simulated predator hung in the test arena.

The simulated predator (plastic dinosaur, Figure 2) was hung halfway from the top of the test arena (88 × 116 × 138 cm) before introducing each chick into the test arena. Once the chick was placed inside the test arena, the door was locked and then the experimenter from outside pulled the rope to which the predator was hung so that it began to swing and the red lights on the simulated predator were lit by pressing a remote. The predator test was undertaken on the 8th, 15th and 22nd days PTH on forty-four YRE and twenty-four FLE chicks. The immediate reaction of each chick was monitored with a CCTV camera positioned inside the test arena for 5 min. The behavior of the chicks was scored on a scale of 1 (not fearful) and 2 (fearful). The behavior of chicks categorized as “not fearful” was when there was no visible change in the chicks' behavior. Chicks were scored as “fearful” when they showed any of freezing, crouching, or running behavior.

### Data analysis

A normality test (Shapiro-Wilk) was performed on the collected data, but none of the data on brooding behavior and fear was normally distributed. So, we used the non-parametric repeated measure analysis, Friedman test, to analyze the behavior of the hens during brooding (three weeks of brooding) and the behavior of their chicks during the tonic immobility test for the three-time points (day 7, 14, and 21 post-hatch). Since the Friedman Test does not allow a between-subject factor (which is ecotype in this case), we analyzed the data using the Friedman test separately for each ecotype and corrected for multiple comparisons using the Bonferroni correction (since we had three timepoints, so significance was based on *P* < 0.017 i.e., 0.05/3 and not on *P* < 0.05). The effect of ecotype on BRD behavior and TI was analyzed using the Mann-Whitney U test at each time point. The behavior of the chicks during the predator test was categorized as either “not fearful” or “fearful”. The effect of ecotype on behavior during the predator test was analyzed using descriptive statistics and inferential statistics. Data from the three body surfaces and egg temperatures were normally distributed and were analyzed using a repeated measures ANOVA having time points (week 1, 2, and 3 post-hatch) as the within-subject factor and ecotype (YRE and FLE) as the between-subject factors. If Mauchly's test of sphericity was significant, then we used Greenhouse Geisser. A Pearson's correlation was undertaken to establish the relationship between the surface body temperatures of the broody hens and the average temperature of their eggs. All statistical analysis was undertaken using the SPSS statistical package (version 23) except for the inferential statistics of the predator test, which was analyzed using the GENMOD procedure of SAS (version 9.4) with binomial distribution and Probit link function.

## Results

### Brooding

#### Behaviors of the hens

**Figure 3:**
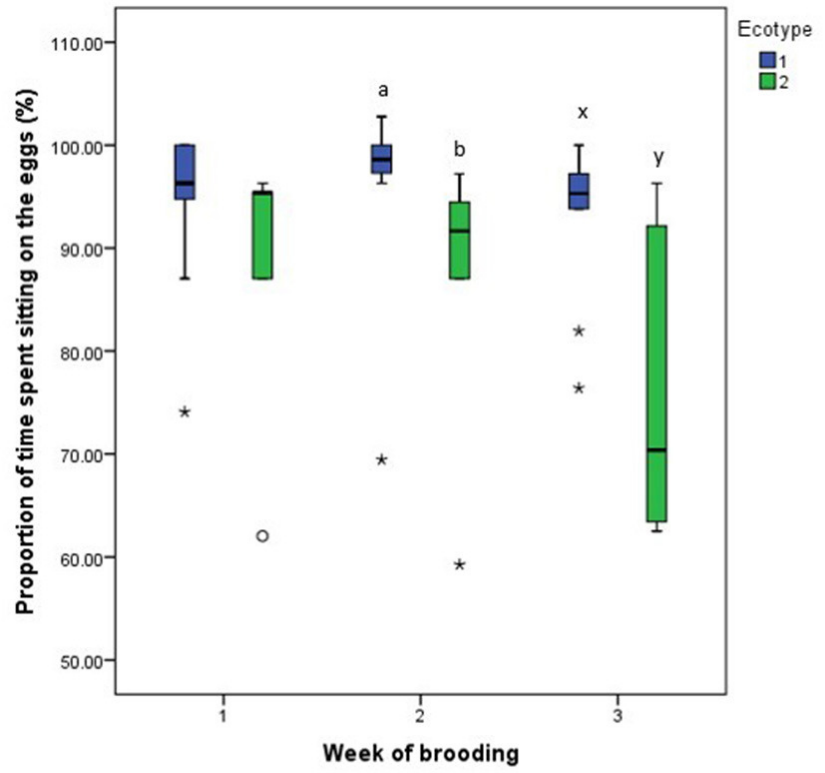
Proportion of time spent by two Nigerian indigenous hen ecotypes (1 = Yoruba and 2 = Fulani) sitting on the eggs over the three weeks of brooding. ^ab^Means differ at *P* < 0.05 at week 2 and ^xy^Means differ at *P* < 0.05 at week 3. Outliers in the data are depicted by the symbol “* or °”.

**Figure 4:**
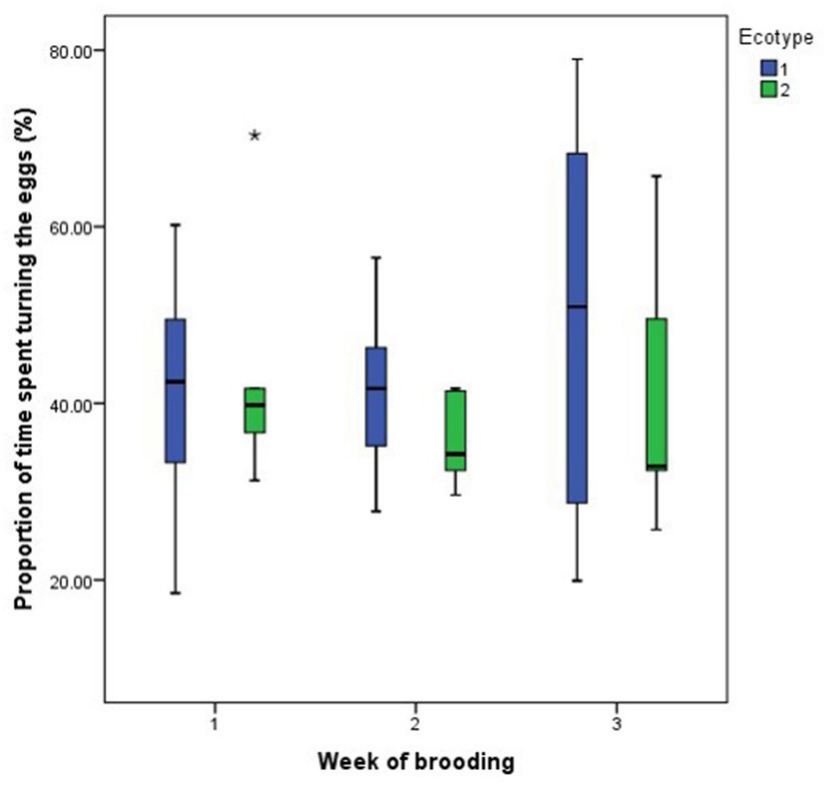
Proportion of time spent by two Nigerian indigenous hen ecotypes (1 = Yoruba and 2 = Fulani) turning the eggs over the three weeks of brooding. Outlier in the data is depicted by the symbol “*”.

**Figure 5:**
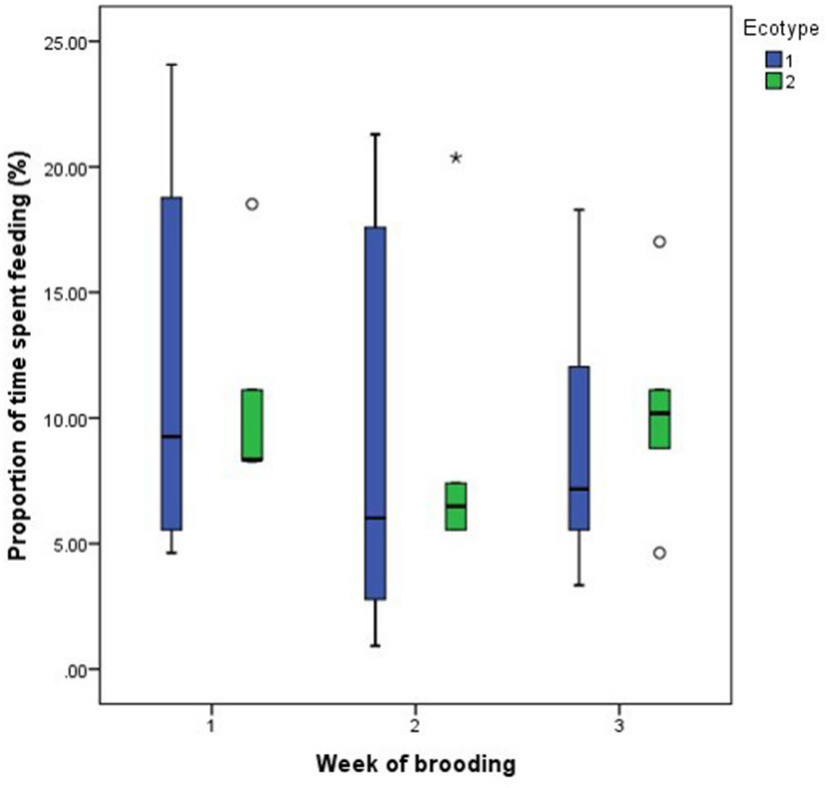
Proportion of time spent by two Nigerian indigenous hen ecotypes (1 = Yoruba and 2 = Fulani) feeding over the three weeks of brooding. Outliers in the data are depicted by the symbol “* or °”.

**Figure 6:**
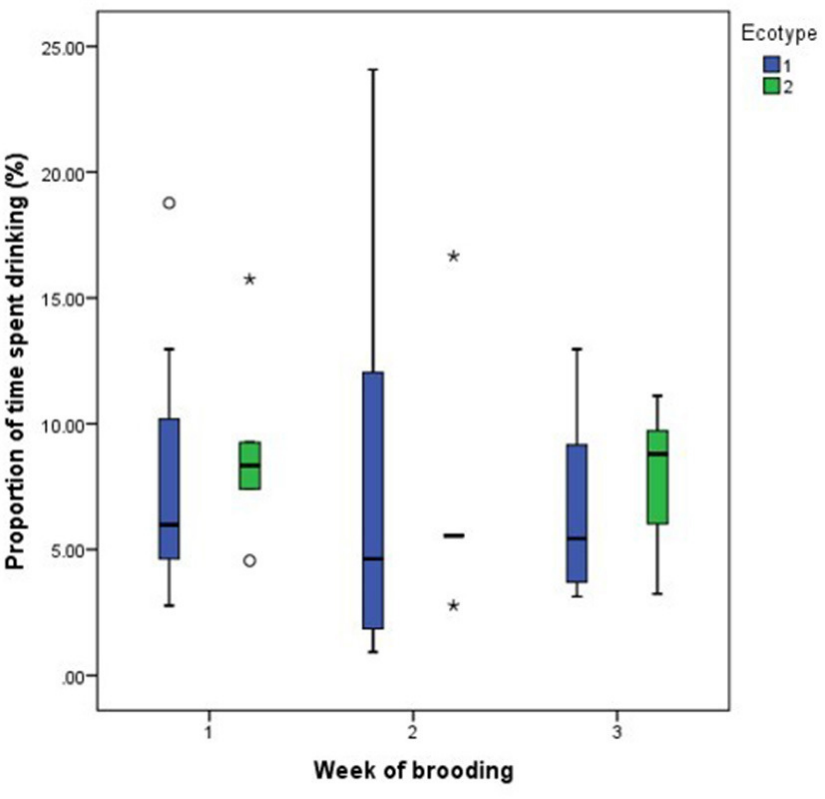
Proportion of time spent by two Nigerian indigenous hen ecotypes (1 = Yoruba and 2 = Fulani) drinking over the three weeks of brooding. Outliers in the data are depicted by the symbol “* or °”.

**Figure 7:**
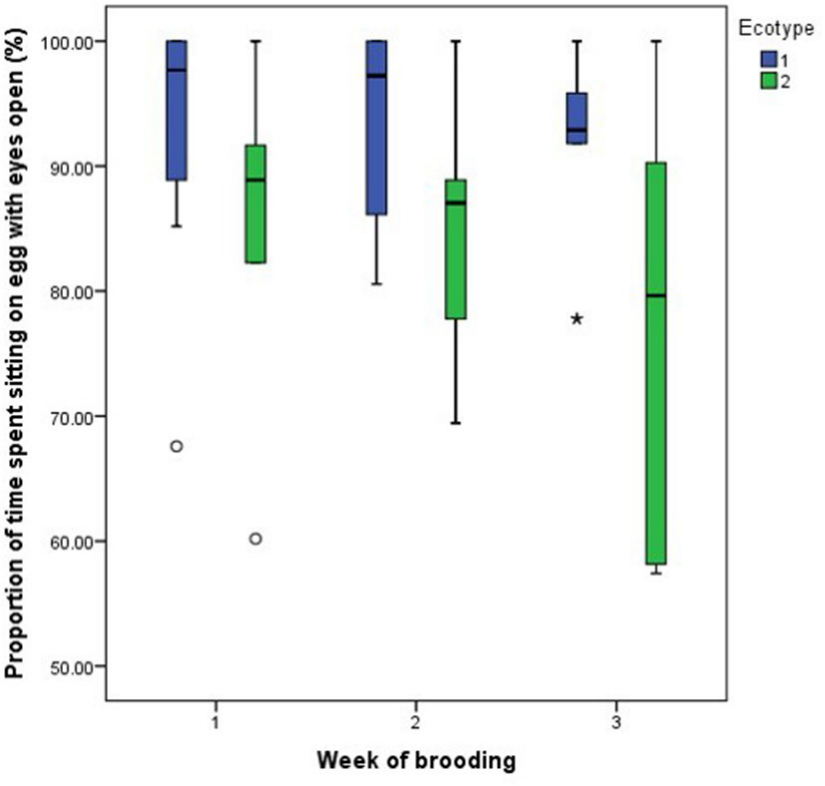
Proportion of time spent by two Nigerian indigenous hen ecotypes (1 = Yoruba and 2 = Fulani) sitting on the egg with eyes open over the three weeks of brooding. Outliers in the data are depicted by the symbol “* or °”.

**Figure 8:**
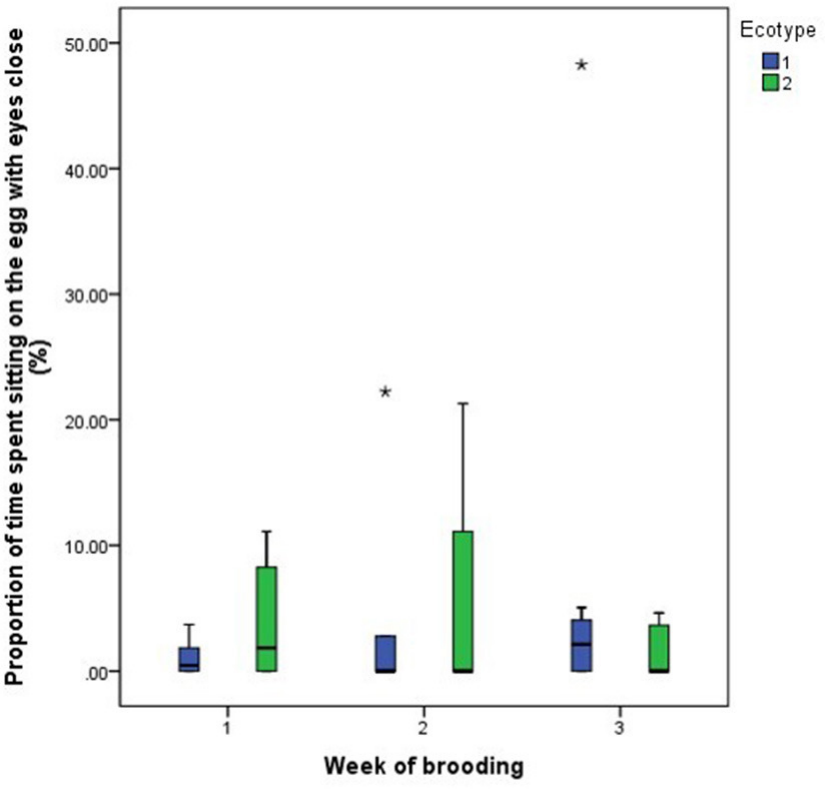
Proportion of time spent by two Nigerian indigenous hen ecotypes (1 = Yoruba and 2 = Fulani) sitting on the eggs with eyes close over the three weeks of brooding. Outliers in the data are depicted by the symbol “*”.

For both hen ecotypes, the proportion of time spent by the hens sitting on the eggs, egg turning, feeding, drinking, and eyes open or close while sitting on the eggs did not differ (*P* > 0.05) over the three weeks of BRD (Figures 3–8). However, the proportion of time spent sitting on the egg was greater in the YRE at the 2nd (*U* = 5.000, *z* = −2.454, *P* = 0.014) and 3rd (*U* = 9.000, *z* = −1.961, *P* = 0.050) weeks of BRD than in FLE hens (Figure 3). Ecotype had no significant effect (*P* > 0.05) on the other BRD behaviors (Figures 4–8).

#### Body surface temperatures of the broody hen

There was no significant effect (*P* > 0.05) of the week of brooding, week × ecotype, and ecotype on the eye, wing, and brood patch temperatures of the two hen ecotypes (Table 2).

**Table 2:** Surface body temperatures of the two ecotypes (Yoruba, YRE, and Fulani, FLE) of broody hens and the average temperature of their eggs for the three weeks of the brooding period.

**Temperatures**	**Week 1**		**Week 2**		**Week 3**	
	**YRE**	**FLE**	**YRE**	**FLE**	**YRE**	**FLE**
Eye temperature (°C)	35.67 ± 0.64	36.23 ± 0.91	35.51 ± 0.53	36.61 ± 0.75	35.94 ± 0.56	36.34 ± 0.80
Wing temperature (°C)	35.67 ± 0.63	36.54 ± 0.89	35.42 ± 0.56	36.75 ± 0.79	35.82 ± 0.67	36.04 ± 0.95
Brood patch temperature (°C)	36.38 ± 0.41	36.82 ± 0.58	36.30 ± 0.38	37.60 ± 0.54	36.70 ± 0.40	37.05 ± 0.57
Egg temperature (°C)	35.51 ± 0.51	35.94 ± 0.73	35.47 ± 0.52	36.13 ± 0.73	35.93 ± 0.52	35.46 ± 0.73

Values are Means ± SEM.

#### Temperature of the brooded eggs

There were no significant effects (*P* > 0.05) of the week, week × ecotype, and ecotype on the temperatures of the broody hens' eggs (Table 2). There were positive correlations (*P* < 0.01) between all three body surface temperatures and the temperature of the brooded eggs (Table 3).

**Table 3:** Pearson's correlation between surface body temperatures of the broody hens and the temperature of the brooded eggs.

	**Eye**	**Wing**	**Brood patch**	**Egg**
	**temperature**	**temperature**	**temperature**	**temperature**
Eye temperature	1.000	0.971^**^	0.881^**^	0.951^**^
Wing temperature		1.000	0.867^**^	0.945^**^
Brood patch temperature			1.000	0.824^**^
Egg temperature				1.000

** P < 0.01.

#### Relative weekly weight loss

There was a significant effect of week of brooding (*F*_1.254,16.302_ = 8.743, *P* = 0.006) on the relative weekly weight loss which was greater at the 3rd than the 1st and 2nd weeks of brooding. There was no significant (*P* > 0.05) week × ecotype interaction and the main effect of ecotype on relative weekly weight loss (Table 4).

**Table 4:** Relative weight loss (%) of the broody hens over the three-week brooding period.

**Ecotype**	**Week 1 (%)**	**Week 2 (%)**	**Week 3 (%)**
Yoruba (YRE), n=10	−1.68 ± 0.18^b^	−2.18 ± 0.20^b^	−3.78 ± 0.64^a^
Fulani (FLE), n=5	−1.73 ± 0.25^b^	−1.60 ± 0.28^b^	−3.44 ± 0.90 ^a^

Values are Means ± SEM,

ab Means differ at P < 0.05.

### Post-hatch fear behaviors in the two chick ecotypes

#### Tonic immobility test

**Figure 9:**
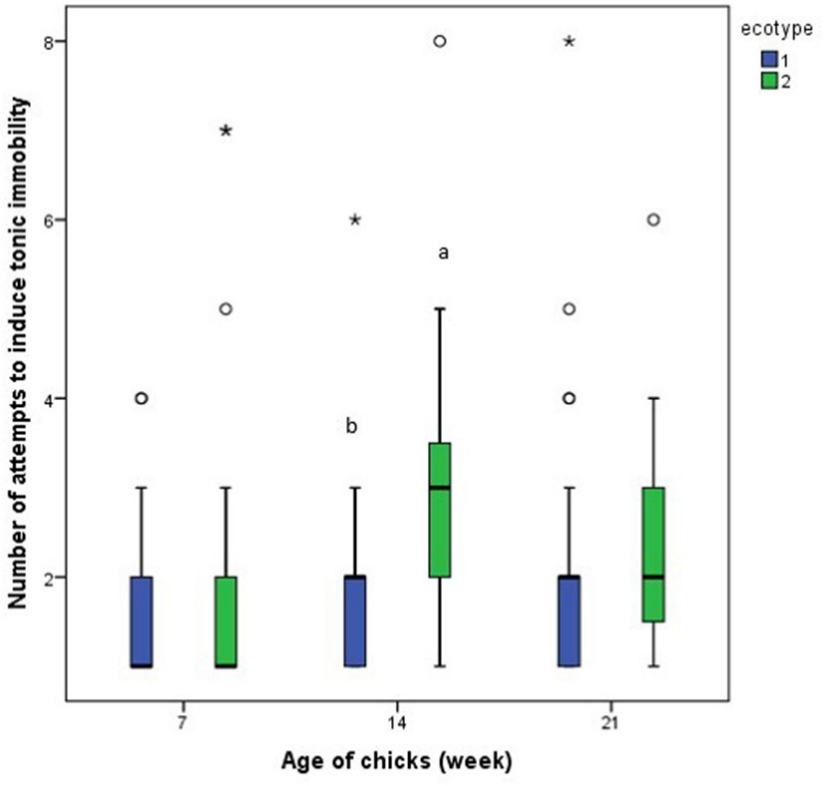
Number of attempts to induce tonic immobility in two ecotypes (1 = Yoruba and 2 = Fulani) of Nigerian indigenous chicks at days 7, 14, and 21 post-hatch. ^ab^Means differ at *P* < 0.05 at day 14 post-hatch. Outliers in the data are depicted by the symbol “* or °”.

**Figure 10:**
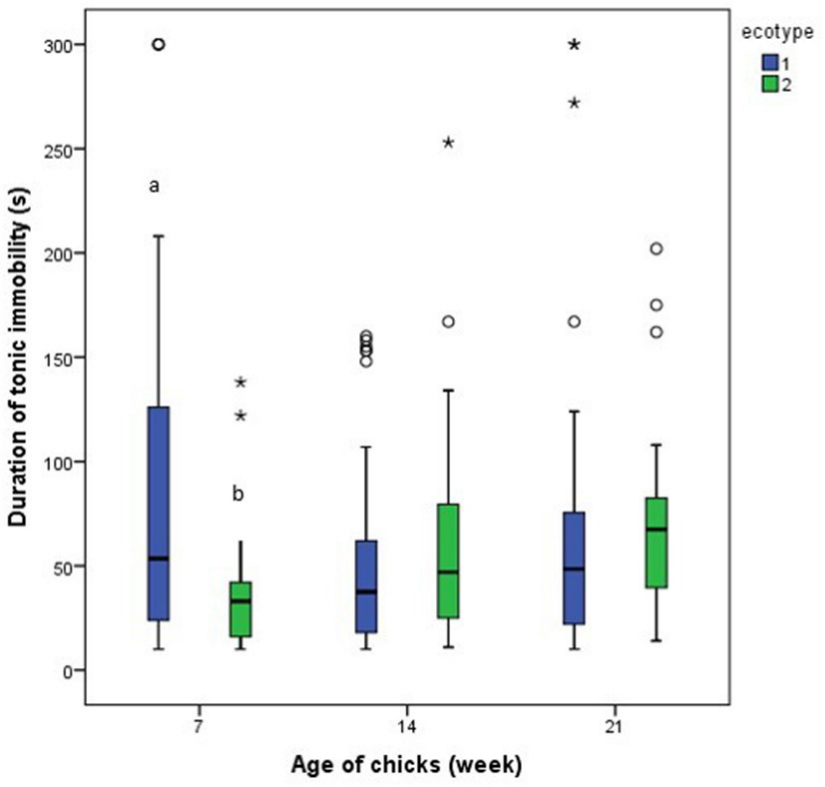
Duration of tonic immobility in two ecotypes (1 = Yoruba and 2 = Fulani) of Nigerian indigenous chicks at days 7, 14, and 21 post-hatch. ^ab^Means differ at *P* < 0.05 at day 7 post-hatch. Outliers in the data are depicted by the symbol “* or °”.

Results from the repeated measures analysis showed that the number of attempts to induce tonic immobility and the duration of tonic immobility for each of the ecotypes was similar across the three-time points (days 7, 14, and 21 PTH), Figures 9, 10. Further analysis of the effect of ecotype showed that on the 14th day PTH, the number of attempts to induce tonic immobility was less (*U* = 332.000, *z* = −2.630, *P* = 0.009, Figure 9) in the YRE than in the FLE chicks. On the 7th-day PTH, the duration of tonic immobility was longer (*U* = 323.000, *z* = −2.632, *P* = 0.008, Figure 10) in the YRE than in the FLE chicks.

#### Predator test

**Figure 11:**
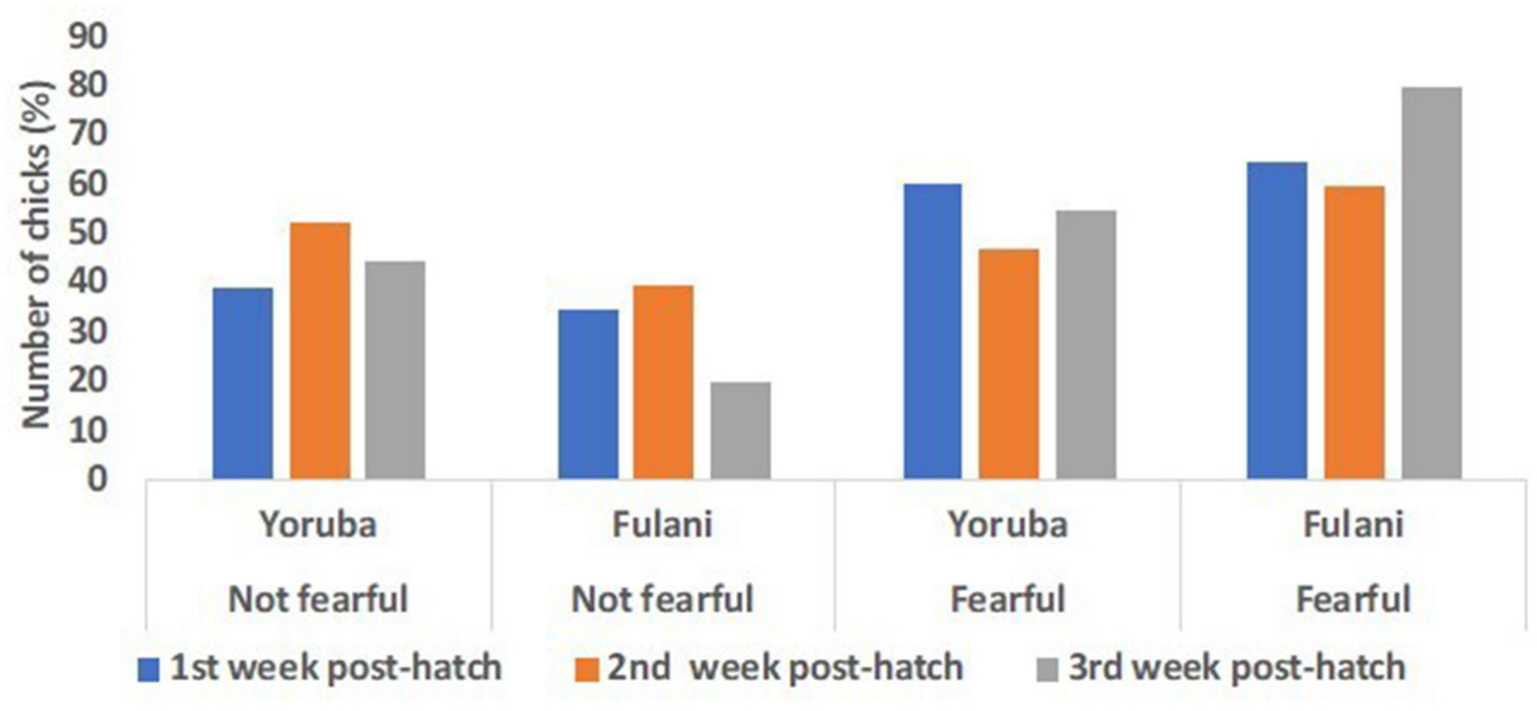
Predator fear score responses in two ecotypes (1 = Yoruba and 2 = Fulani) of Nigerian indigenous chicks at weeks 1–3 (i.e., day 8 (blue bars), day 15 (orange bars), and day 22 (gray bars) post-hatch).

Although the inferential statistics revealed no effect of ecotype on the fear score on the 8th, 15th, and 22nd PTH days, the descriptive statistics show some interesting trends (Figure 11). The result showed two distinct fear responses in the chicks: the “not fearful” and the “fearful” categories. The percentage of chicks that showed no fear response to the simulated predator was similar in the two ecotypes on the 8th day PTH, but on the 15th and 22nd -day PTH, the percentage of YRE chicks seemed to increase and seemed to be greater than the FLE chicks. On the other hand, among the chicks that showed a higher fear response to the simulated predator (fearful category), on the 8th-day PTH, there was a similar percentage of YRE and FLE chicks. However, on the 15th and 22nd days PTH, the percentage of YRE chicks was reduced and the FLE chicks increased. Overall, there seemed to be a greater number of chicks that belonged to the “fearful” category than to the “not fearful” category.

## Discussion

The aim of this study was to investigate the potential of having a tropically adapted chicken breed with high productivity to meet the protein needs of the Nigerian population and have good maternal care to raise offspring with fewer welfare issues and the ability to survive in a free-range rearing system based on their ability to display appropriate fear responses when they encounter real-time predators.

It was our intention to have a minimum of 15 broody hens per ecotype, but within the 7-month experimental period (July 2021 and January 2022), only 10 out of 30 (33.3%) YRE and 5 out of 30 (16.7%) FLE hens became broody. A previous study by Iyasere et al. (17) on BRD behavior, reported a 30% success of the YRE becoming broody in a similar intensive rearing system. The breakdown of when the hens became broody in the current study is as follows; July (three YRE and one FLE), September (three YRE), October (two YRE and one FLE), November (two YRE), and December (three FLE). The reason for the low number of FLE hens that became broody within the period of this study could indicate that they needed more time to get acclimatized to the intensive conditions at our research station. We sourced the FLE chickens from Fulani people that settled in a village in Kishi, Irepo Local Government Area of Oyo State, where they are raised under the extensive system. There could also be the possibility of the season affecting the broodiness of the FLE hens because three out of the five FLE hens that became broody were recorded in December which falls in the early dry season of the year in Nigeria. The FLE chickens originated from Northern Nigeria, so their breeding season may be favored by hot or dry weather. Further studies are required to investigate the influence of acclimatization and season on broody hens in these two chicken ecotypes.

It is also worth mentioning that the low number of broody hens could be due to the fact that we adopted a natural broody method in the current study where eggs were left in the nest boxes and hens were exposed to natural daylight (12L:12D). Other studies have induced broodiness by extending the daylight to 16 h in addition to the provision of eggs in the nest box, which resulted in a 46.7% success in the Silkie and Wyandotte hens (23, 30). In order to increase the number of broody hens in future studies, we may consider the extension of daylight after first investigating whether induction has no negative welfare implications for the hen.

In the current study, each ecotype showed no difference in all the BRD behaviors monitored over the three weeks. This implies that once BRD commenced, the hens' behaviors remained consistent irrespective of the stage of development of the embryo, until the chicks hatch. Iyasere et al. (17) also observed consistent sitting on eggs and ingestive behavior in the YRE ecotype over the three weeks of BRD. Broodiness is controlled by the prolactin hormone (31). Behaviorally, the most obvious sign of BRD in a hen is continuous sitting in the nest box whether on eggs or not, and emitting a “growling sound” and puffing of feathers when approached. Other behavioral changes include reduced feed and water intake, turning and retrieval of eggs, aggressive or defensive behaviors, and cessation of egg-laying (32). As heat is transferred from the hen through the brood patch to the eggs for the development of the embryo, it is very important that the hen turns the egg at intervals to ensure uniform development of the embryo and prevention of embryo from sticking to the shell (33).

The reduction in feeding activities during BRD causes the hens to lose weight. The higher relative weight loss in both hen ecotypes at the 3rd week of BRD could be attributed to a greater depletion of body reserves required to maintain the heat production needed for the development of the embryo (34). We observed a 7.64 and 7.21% relative weight loss over the three weeks in the YRE and FLE ecotypes, respectively. Brooding pheasant hens lose weight from almost all body tissues and organs (35).

In the current study, both hen ecotypes were provided with ten eggs each to incubate once broodiness was confirmed. The longer time spent sitting on the eggs by the YRE hens in the 2nd and 3rd weeks could be due to two main reasons. Firstly, the YRE hens may need extra effort to accommodate the large number of eggs placed underneath them since they have smaller chest dimensions, which easily accommodate their small clutch size of 2–6 eggs, compared to those of the FLE hens, with bigger chest dimensions to accommodate a bigger clutch size of 3–9 eggs (14). Secondly, the YRE eggs have a thicker eggshell (5.12 mm) compared to the FLE (4.89 mm) eggs (14), so more effort may be required from the hen to generate the needed heat to penetrate this thick shell for the development of the embryo.

In a comparative study on the effect of body size of Bangladesh broody hens on hatchability and chick survival, it was observed that Bangladesh broody hens with an average body size of 800–950 g were able to hatch 87.2% of the eggs when provided with 17 eggs to incubate, each with an average weight of 41 g (36). However, there was no report on whether the body size of the hens influenced their BRD behavior. The inability of the hens to hatch the remaining 12.8% could be that their small body size could not accommodate all the eggs underneath them. For a hen with a body size of 800–950 g, Azharul et al. (36) recommended placing 14 eggs for incubation. The YRE hens used in the current study have an average body weight of 828 g, which is close to that reported in the Bangladesh broody hens, so the YRE may not have the capacity to incubate as many eggs as the Bangladesh hens.

Broody hens sit on their eggs to provide the heat which is transferred from their bodies, especially the chest/breast region or brood patch, to the eggs. This corroborates our observation of both ecotypes having similar SBTs (eye, wing, and brood patch) and EGT, as the developing embryo is very sensitive to temperature changes. Interestingly, we observed positive correlations between the SBTs of the hen and the temperature of the eggs she was brooding. In addition, the current study showed that the SBTs of the hens of both ecotypes were similar over the three-week BRD period. This implies that the hens were able to maintain their body temperatures at a level that was appropriate for the development of the embryo. Iyasere et al. (17) previously reported that the rectal temperature of the YRE hens remained constant over the three weeks of BRD, but the breast temperature was higher during the first and second weeks than during the third week of BRD. The reason for this inconsistency could be related to the robust data available in the current study (hens' SBTs were measured three times a week and the average calculated per week), but a single measurement per week was taken in the study of Iyasere et al. (17).

In this study, we made use of tests that have been validated in chickens as a measure of fear. The open field test was not undertaken because the response of animals in this test is a combination of two motivations: fear and the need for social reinstatement (37). We adopted the TI test as a measure of the level of fear in the current study because TI is an anti-predator freezing response (feigning death) in which prey species adopt a relatively immobile state that can last from seconds to hours after the physical restraint has ceased (38–40). The TI can function to reduce the perceived need of the predator to further subdue the prey, thereby increasing opportunities for the prey to escape and survive (40–42). A predator model is an established method to score individual variations in fear (37).

The YRE and FLE showed consistency in the number of attempts to induce TI and the duration of TI over the three testing time points. This implies a stable fear response over the first three weeks of life, which happens to be the most critical point contributing to their survivability. In addition, testing the chicks once a week for three weeks did not induce any form of habituation. Studies have reported that chicks get accustomed to TI, showing reduced susceptibility and duration to TI when they are subjected to repeated daily testing (43, 44).

From the behavioral responses of the chicks to the simulated predator test, we observed that a higher percentage of the chicks of both ecotypes seem to belong to the “fearful” category. This suggests that the chicks perceived the simulated predator as a potential one and adopted behaviors such as freezing, crouching, or running to escape from it. From an evolutionary point of view, these behaviors may enhance fitness and survival in the wild (37). From the TI and simulated predator tests used in the current study, the YRE chicks were more fearful, having a longer duration of TI on day 7 PTH, and they easily entered TI on day 14 PTH. Despite this interesting finding in the differences between the ecotypes, it is worth mentioning that the interpretations of fear responses and their implications on welfare seem to be context-dependent. The display of a high level of fear in birds housed in an intensive system may be considered counterproductive as this could result in piling and smothering leading to injury and even death. However, in the natural environment (wild) or for birds that are considered for free range systems, the birds need to show appropriate behavioral responses, which endows them with better fitness and survivability.

Based on the variability in the fear responses of the chicks of the two ecotypes, we can suggest that the two ecotypes can be considered as chicken ecotypes suitable for different housing systems; the YRE for an outdoor/free range system because of their ability to escape from predators by displaying a high level of fear; and the FLE for an indoor production system. However, further studies would be required to validate this, as Lindholm et al. (45) reported that longer tonic immobility observed in the slow-growing broiler strain (Rowan Ranger) did not affect their use of the range. The level of fear appears to be influenced by body weight. The increased level of fear in the YRE could also be related to the lower body weight compared to the FLE chicks. Further studies on the influence of age and body weight on the level of fear experienced by these two ecotypes will be needed.

## Conclusion

This study observed some influence of ecotype on maternal behaviors of Nigerian indigenous hens during brooding and the level of fear of their chicks. The YRE hens spent more time sitting on the eggs. The FLE chicks had a lower level of fear in the TI test but showed a higher fear response to simulated predator attack, which is needed in case the bird is exposed to a real-life predator. Results from this study show that the FLE hens can be recommended as “ecotype with good welfare” with better feed conversion and produce more meat and eggs to meet the nutritional requirements of man and have economic benefits to the rural poor farmers. The welfare of the chicks in terms of fear and behavioral responses to escape from predators could be a potential criterion that can be used to determine the best housing system for the ecotypes. The YRE ecotype showing higher predator escape behavior may be considered for free-range housing production because this behavior can enhance their survivability in the natural environment when faced with real-time predators.

We therefore recommend an improvement in both ecotypes using the appropriate breeding programs that would improve the productivity (feed conversion, meat and egg) of FLE in an intensive management system, and broodiness as well as survivability of YRE under an extensive system.

## Data availability statement

The original contributions presented in the study are included in the article/supplementary material, further inquiries can be directed to the corresponding author/s.

## Ethics statement

All procedures of this study were based on guidelines of the Animal Care and Use Committee of the Federal University of Agriculture, Abeokuta, Ogun State Nigeria.

## Author contributions

VO, OI, SD, and JD were involved in the conceptualization, design, implementation, and manuscript writing. FF, POdeta, SO, POdetu, and TA were involved in fieldwork, data collection, and extraction of behavioral data from the video. All authors contributed to the article and approved the submitted version.

## Funding

This research was funded by the Universities Federation for Animal Welfare through the student scholarship scheme.

## Conflict of interest

The authors declare that the research was conducted in the absence of any commercial or financial relationships that could be construed as a potential conflict of interest.

## Publisher's note

All claims expressed in this article are solely those of the authors and do not necessarily represent those of their affiliated organizations, or those of the publisher, the editors and the reviewers. Any product that may be evaluated in this article, or claim that may be made by its manufacturer, is not guaranteed or endorsed by the publisher.
